# Effect of Ionic Polymer Membrane with Multiwalled Carbon Nanotubes on the Mechanical Performance of Ionic Electroactive Polymer Actuators

**DOI:** 10.3390/polym12020396

**Published:** 2020-02-10

**Authors:** Joohee Kim, Minjeong Park, Seonpil Kim, Minhyon Jeon

**Affiliations:** 1 Department of Nanoscience and Engineering, Center for Nano Manufacturing, Inje University, Gimhae 50834, Korea; 21wngml@gmail.com (J.K.); mjpark9121@gmail.com (M.P.); 2 Department of Military Information Science, Gyeongju University, Gyeongju 38065, Korea; seonpil@gu.ac.kr

**Keywords:** ionic electroactive polymer, Nafion membrane, multiwalled carbon nanotubes, heat press two-step process, blocking force

## Abstract

Ionic electroactive polymer (IEAP) actuators have received interest because of their advantageous properties, including their large displacement, high energy density, light weight, and low power consumption under a low electric field. However, they have a low blocking force under driving, and it is difficult to control the thickness of the ionic polymer membrane. In this study, an IEAP actuator is fabricated using a Nafion membrane with added multiwalled carbon nanotubes to increase the blocking force. A heat press two-step process is also developed to produce a constant and uniform membrane. The fabricated Nafion membrane with 0.2 wt% multiwalled carbon nanotubes has the largest displacement and highest blocking force. As a result, the developed heat press two-step method can be used in various polymer-casting fields, and the fabricated carbon nanotube-based IEAP actuators can serve as useful references in fields such as flexible robotics and artificial muscles.

## 1. Introduction

The advantageous properties of ionic electroactive polymer (IEAP) actuators include their large displacement, high energy density, light weight, and low power consumption under low electric fields [1,2,3,4]. Typically, IEAP actuators are composed of an ionic polymer membrane sandwiched between two noble metal electrodes [5,6], and the diffusion of hydrated cations within the membrane under an applied voltage and the associated electrostatic interactions induce bending.

Nafion membranes have excellent ionic conductivity and chemical and thermal stability, and they are suitable up to a thickness of 180 μm for application as ionic membranes in IEAP actuators [7]. Commonly, IEAP actuators are limited by the commercially available Nafion thicknesses (e.g., N115, N117, and N1110), and thus various studies on controlling their thickness using a Nafion solution have been reported [6,8,9,10,11,12,13,14,15,16,17]. Furthermore, since the thickness of the membrane significantly affects its properties and experimental behavior, it is highly important to fabricate consistent and uniform membranes like those that are commercially available membranes in order to compare and verify experimental results. For these reasons, *N*,*N*′-dimethylformamide (DMF) was used to cast the Nafion membranes in this study. DMF is highly compatible with the Nafion tetrafluoroethylene backbone, and thus enables the formation of a membrane in which the Nafion molecules do not aggregate [17]. It is also used as a solution for carbon nanotube (CNT) dispersions such as for surfactants [18,19,20]. However, in the Nafion membrane casting process, it proved to be difficult to acquire a uniform membrane with a controlled thickness. So, after the casting process, a new method of controlling the thickness through additional processes was needed. The heat press is good because it can process heat and pressure at the same time, but the fine and constant thickness control method needs to be further investigated. Therefore, the process using the metal plate and the heat press was further performed after the casting to obtain a uniform membrane having a controlled thickness.

Moreover, IEAP actuators have significant drawbacks such as low actuation bandwidth, low blocking force, and poor durability [21,22,23,24]. Their low blocking force has particularly hindered the practical applicability of IEAP actuators, necessitating studies on increasing their blocking force. The thicker membrane layer or the thicker electrode layer can cause a displacement decrease of the actuator [8,9,10], and the stiffer electrode layer can cause cracks on the electrode surface, and poor durability of the actuator in operation. Therefore, the application of various materials such as graphene, fullerene, carbon-metals, and graphene oxide to the actuator is studied to increase blocking force or improve actuation performances without reducing displacement [11,12,13,14,15,16,25,26,27,28]. Among various proposed additive materials, multiwalled carbon nanotubes (MWCNTs) have shown high chemical and thermal stability, excellent tensile strength and stiffness, and high conductivity and heat resistance. The excellent properties of the MWCNTs improve the mechanical, electrical, and thermal properties of various polymers [29,30,31,32]. The MWCNT was used to increase the blocking force while maintaining the displacement. However, acid treatments are commonly required in the dispersing process; these treatments damage actuators and affect their mechanical properties owing to the resulting CNT structural defects, which are undesirable for further actuator applications [33]. In contrast, Nafion can be used as an alternative to acid treatment to overcome the disadvantages arising from the chemical functionalization and physical dispersion of CNTs. Furthermore, dispersion occurs due to hydrophobic interactions between Nafion and CNTs [20], and thus MWCNTs have been considered as additives in Nafion membranes [23,24,34]. The resulting Nafion membranes with MWCNTs (N-MWCNT) are easily fabricated and have tunable stiffness and high ion transport. N-MWCNT also have the potential to enhance the blocking force and response speed of IEAP actuators.

Since IEAP is flexible, small, and lightweight, it is mainly developed for miniaturized and lightweight robots. Lee and Jain studied the movement of artificial muscle fingers using EMG signals, respectively [35,36]. As well as artificial human muscle simulation, much research has been done on robots as well as artificial human muscle simulation that simulates aquatic life such as jellyfish and fish [37,38,39]. Thus, the driving force is an essential factor in developing the application as an artificial muscle of a living organism.

In this study, we developed a heat press two-step (HPTS) process to obtain Nafion membranes with uniform thicknesses and fabricated N-MWCNT-based IEAP actuators. We prepared N-MWCNT membranes with a thickness of 200 μm by the developed HPTS process accounting for the fact that the membrane thickness was reduced by approximately 10% when the actuators were manufactured with the paper electrodes [7]. We created N-MWCNT membranes with various MWCNT weight ratios for IEAP actuators and observed their thermal and mechanical properties.

## 2. Experimental

### 2.1. Materials

DMF was purchased from Sigma-Aldrich (St. Louis, MI, U.S.), and MWCNTs were purchased from Carbon Nanomaterial Technology Co., Ltd. (Pohang, Gyeongbok, South Korea). A Nafion solution (20 wt%; D2021) was purchased from Dupont (St. Wilmingtoon, Delaware, U.S.), and Nafion membranes were cast using mixtures of the Nafion solution (16 g), DMF (5 wt%), and MWCNTs (0.0~1.0 wt%). Graphene oxide (GO) and a silver nanowires (Ag NWs) solution were purchased from Grapheneall Co., Ltd. (Siheung-si, Gyeonggi-do, South Korea) and DUKSAN Hi-Metal (Ulsan, Gyeongnam, South Korea), respectively, and electrodes were used. Lithium chloride purchased from Sigma-Aldrich (St. Louis, MI, U.S.) and 1-ethyl-3-methylimidazolium trifluoro-methyl-sulfonate (ionic liquid) purchased from Merck KGaA (Darmstadt, Hesse, Germany) were used for actuator ion exchange.

### 2.2. Fabrication of N-MWCNT Membranes

**Figure 1:**
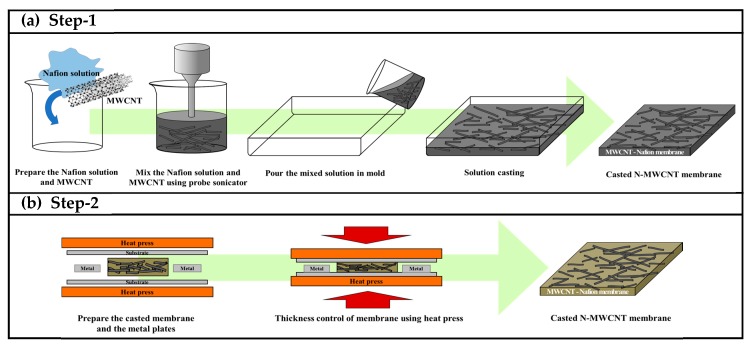
Schematics diagram of Nafion membranes with MWCNTs (N-MWCNT) membrane fabrication process by heat press two-step (HPTS) method (**a**) step-1 and (**b**) step-2.

The developed HPTS method employs a hot press and metal plate in two steps. Figure 1a shows step 1, in which the approximate film thickness was determined by the weight of the dispersion. Nafion solutions containing DMF and MWCNTs were mixed using a probe sonicator, and the mixture was poured into a preheated casting mold (9.0 × 9.0 cm^2^). The filled mold was heated at 40 °C for over 12 h to vaporize the volatile components of the Nafion solution (deionized water and acetone) and thus obtain the casted N-MWCNT membrane. Next, in step 2 (Figure 1b), the casted N-MWCNT membrane from step 1 and support metal plates of the desired thickness were placed between substrates with high heat resistance and strength and were heat-pressed at 140 °C and 10 MPa. Herein, the metal plate that was used was not deformed by heat and pressure, had a clean surface, and was thinner than the membrane cast in step 1. Conditions of the process such as time, temperature and pressure were optimized through repeated experiments. Finally, we obtained N-MWCNT membranes with uniform thicknesses by the HPTS method.

### 2.3. Fabrication of N-MWCNT-Based Actuators

Actuators were manufactured using the N-MWCNT membranes and GO-Ag NWs electrodes. We used the GO-Ag NWs (weight ratio 1:2.5) paper electrodes that Yoo, S. studied [40], which were made using a vacuum filtration system. Each N-MWCNT membrane fabricated by the HPTS method was placed between the GO-Ag NWs paper electrodes and attached using the heat press to yield the N-MWCNT-membrane-based actuator. The size of fabricated actuator was 4.0 × 0.5 cm^2^.

### 2.4. Analytical Techniques

We created N-MWCNT membranes with various MWCNT weight ratios for IEAP actuators and observed surface morphologies and thicknesses using field emission scanning electron microscopy (FE-SEM; S-4300, Hitachi, Tokyo, Japan) and a Micrometer analyzer. The thermal stabilities of the fabricated membranes were assessed by thermo-gravimetric analysis (TGA), and the mechanical performances of the N-MWCNT-based IEAP actuators were analyzed using a driving characteristics analyzer and tensile strength machine. The actuation performances of the actuators were measured using a laser displacement sensor (ZS-LD80, OMRON Korea, Seocho, Seoul, South Korea) and load cell sensor. The displacement was analyzed to the changing distance of the laser on the surface of the actuator when AC voltage was applied to the actuator. The blocking force was analyzed to the contacting load cell sensor with the surface of the actuator when DC voltage was applied to the driver.

## 3. Results

**Figure 2:**
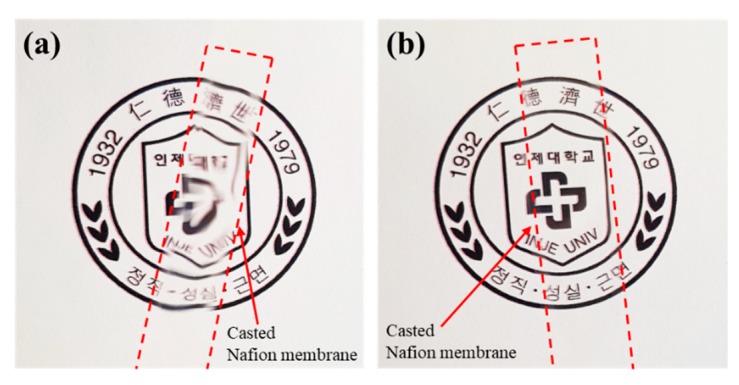
Photographs of Nafion membranes fabricated by (**a**) evaporation method and (**b**) HPTS method.

Nafion membranes for use as ionic polymer membranes in actuators were made using evaporation and HPTS methods, and were compared. As shown in Figure 2a, the surface of the Nafion membrane made by evaporation method was not flat or uniform, and it is clear that the image behind the Nafion membrane is distorted. In contrast, the surface of the Nafion membrane made by HPTS method was smooth; the image is clear, well transmitted, and not distorted (Figure 2b).

**Figure 3:**
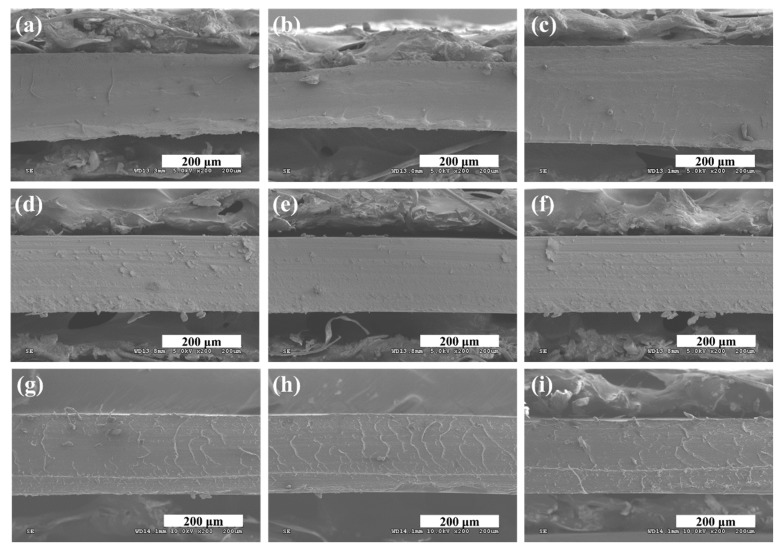
Cross-sectional SEM images of (**a**–**c**) evaporation-fabricated Nafion membrane without multiwalled carbon nanotubes (MWCNT), (**d**–**f**) HPTS-fabricated Nafion membrane without MWCNT, and (**g**–**i**) HPTS-fabricated Nafion and 1.0 wt% MWCNT composite membrane.

**Figure 4:**
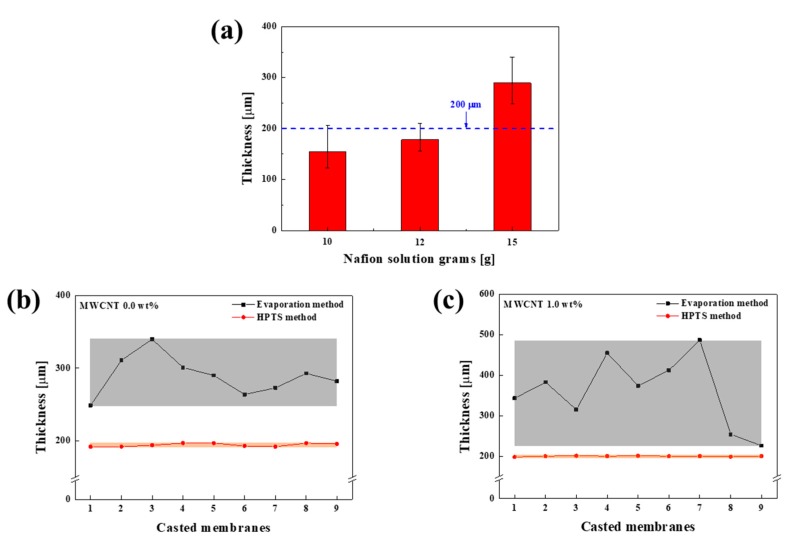
Thicknesses of casted Nafion membranes: (**a**) fabricated by evaporation method with different amounts of Nafion solution (10, 12, and 15 g); fabricated by evaporation (black) and HPTS (red) (**b**) with a 15 g Nafion solution and (**c**) with 1.0 wt% MWCNTs added to a 15 g Nafion solution.

Figure 3 shows the SEM cross-sectional images of the Nafion membrane fabricated with 15 g of the Nafion solution measured at three different areas. Figure 4a shows the average thicknesses and standard deviations of the membranes fabricated by evaporation method according the amount of Nafion solution (10, 12, and 15 g). The membranes fabricated by evaporation did not have uniform thicknesses and also showed large thickness variations between samples with the same weights of added Nafion. Therefore, it was not possible to obtain the desired membrane thickness. Herein, because the required Nafion membrane thickness was 200 µm, we manufactured the membrane using a 15 g Nafion solution having the sufficient thickness to which the pressing process can be applied. Figure 3a–c shows SEM cross-sectional images of the evaporation-fabricated Nafion membrane, and Figure 3d–f shows those of the HPTS-fabricated membrane. It is clear from the images that the thickness of the evaporation-fabricated membrane was nonuniform, whereas the HPTS-fabricated membrane exhibited a uniform thickness. Figure 3g–i shows SEM cross-sectional images of the HPTS Nafion membrane fabricated with MWCNTs at 1.0 wt%, which also had a uniform thickness.

Figure 4b,c shows the thicknesses of the Nafion membranes fabricated using a 15 g Nafion solution and N-MWCNT 1.0 wt% by both evaporation and HPTS methods. The thickness deviations derived from nine experiments of the evaporation-fabricated Nafion and N-MWCNT 1.0 wt% membranes were 18.2 and 87 times larger, respectively, than those of the corresponding HPTS-fabricated membranes. Despite the addition of MWCNTs, the casted HPTS Nafion membranes had uniform thicknesses of 200 μm. These results show that our HPTS method is more suitable than the evaporation method for producing uniform Nafion membranes and is extensively applicable to various other efforts, such as controlling the thickness of polymers.

**Figure 5:**
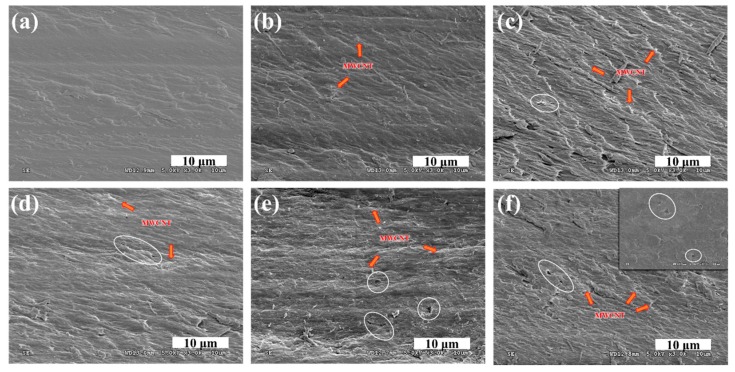
Cross-sectional SEM images of N-MWCNT membranes with different MWCNT weight ratios: (**a**) 0.0 wt%, (**b**) 0.2 wt%, (**c**) 0.4 wt%, (**d**) 0.6 wt%, (**e**) 0.8 wt%, and (**f**) 1.0 wt%.

Figure 5 shows cross-sectional SEM images of the HPTS N-MWCNT membranes. The N-MWCNT 1.0 wt% membrane had an aggregated surface (Figure 5f), and thus we fabricated N-MWCNT membranes with different MWCNT weight ratios from 0.0 to 1.0 wt% and compared their properties. The SEM images show that MWCNT and Nafion were evenly mixed in general, but a greater MWCNT weight ratio led to a partially aggregated surface (the white circles in the figure). The Nafion membrane made with MWCNT at 0.2 wt% (N-MWCNT 0.2 wt%) had the smoothest surface and showed no aggregation. This result implies that 0.2 wt% MWCNTs is the most suitable amount for achieving a good dispersion in the Nafion solution.

**Figure 6:**
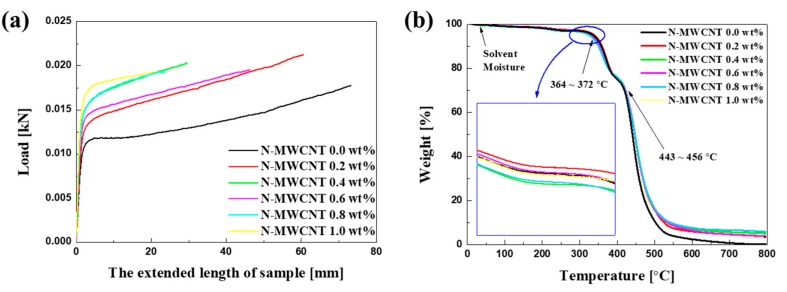
(**a**) Tensile moduli and (**b**) thermo-gravimetric analysis (TGA) curves of casted HPTS Nafion membrane without MWCNTs and N-MWCNT membranes with different MWCNT weight ratios (inset: enlargement of 364–372 °C TGA range).

The tensile strengths of the Nafion and N-MWCNT membranes (Figure 6a) were also analyzed. The tensile strength of the N-MWCNT 0.2 wt% membrane was 22.3 MPa, which was 1.19 times higher than that of the Nafion membrane without MWCNTs. The N-MWCNT 0.2 wt% membrane also had the highest tensile strength of all the N-MWCNT membranes, owing to its nonaggregated surface.

The obtained TGA results indicate the thermal stability of the Nafion and N-MWCNT membranes (Figure 6b). Weight loss occurred in all the Nafion membranes between approximately 370 and 450 °C. The initial weight loss above 300 °C could be due to the decomposition of sulfonic acid groups, and the remaining majority of the weight loss is due to decomposition of carbon-fluorine bonds [41]. The N-MWCNT 0.2 wt% membrane had a higher thermal stability than both the Nafion membrane without MWCNTs and the other N-MWCNT membranes (inset of Figure 6b). These results show that the thermal stability of the N-MWCNT 0.2 wt% membrane was improved because the MWCNTs were well dispersed in the Nafion membrane.

**Figure 7:**
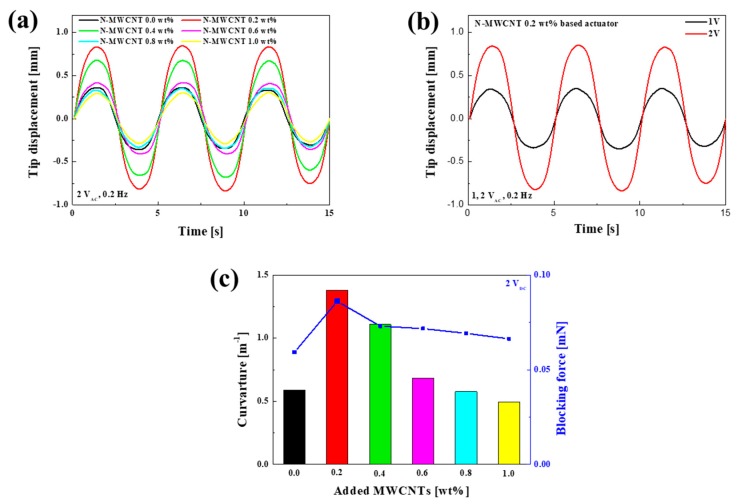
(**a**) Tip displacements of casted Nafion-based actuator and N-MWCNT-based actuators with different MWCNT weight ratios under 2 V_AC_ and 0.2 Hz, (**b**) tip displacements (1 and 2 V_AC_, 0.2 Hz) of N-MWCNT 0.2 wt%-based actuator, and (**c**) curvatures and blocking forces of casted Nafion-based actuator and N-MWCNT-based actuators with different MWCNT weight ratios under 2 V_DC_.

The GO-Ag NWs paper electrode that we used had an electrical conductivity (σ) of about 9615 S/cm, a sheet resistance of about 250 mΩ/sq., and a thickness of about 14 µm. The actuator was fabricated with the N-MWCNT membrane. Figure 7 shows the actuation performances of the Nafion-based actuator without MWCNTs and the N-MWCNT-based actuators. The N-MWCNT 0.2 wt%-based actuator featured a displacement of 0.845 mm, which was 2.34 times larger than that of the Nafion-based actuator without MWCNTs (Figure 7a). We also observed that the maximum displacement decreased as the MWCNT weight ratio increased. Figure 7b shows the actuation performances of the N-MWCNT 0.2 wt%-based actuator under various input voltages (1 and 2 V_AC_) at 0.2 Hz. The displacement of the N-MWCNT 0.2 wt%-based actuator decreased but still stably actuated under 1 V_AC_. Figure 7c shows the bending curvatures and blocking forces of the casted Nafion-based actuator and N-MWCNT-based actuators with different MWCNT weight ratios under 2 V_DC_. The bending curvatures were calculated using Equation (1):

(1) κ=1/R=2δ/(l^2+δ^2)

where R, δ, and l are the radius of curvature, tip displacement, and actuator free length, respectively. The free length in the actuation measurement is the length from the position of the electrode in contact for voltage application to the laser point measuring the displacement with actuator surface standard. The N-MWCNT 0.2 wt%-based actuator had the highest blocking force of 0.086 mN, which was 1.6 times larger than that of the Nafion-based actuator without MWCNTs (Figure 7c). These results show that a high actuator performance resulted from the addition of MWCNTs, yielding a Nafion membrane with high strength and thermal stability. Herein, we observed that the N-MWCNT 0.2 wt%-based actuator had the best actuation performance compared with the Nafion-based actuator and other N-MWCNT-based actuators.

## 4. Conclusions

In this study, we developed an HPTS method to produce Nafion membranes with consistent and uniform thicknesses, and we fabricated N-MWCNT membranes using different MWCNT weight ratios to increase the actuator blocking force. The deviations in thickness of the HPTS-fabricated Nafion and N-MWCNT membranes were 18.2 and 87 times lower, respectively, than those of the corresponding membranes fabricated by evaporation method. Therefore, the Nafion and N-MWCNT membranes fabricated by the HPTS method had highly uniform thicknesses and surfaces. We determined the suitable MWCNT weight ratio to be approximately 0.2 wt% for application to IEAP actuators. The N-MWCNT 0.2 wt%-based actuator had a tensile strength of approximately 22.3 MPa, which was 1.19 times higher than that of the Nafion-based actuator without MWCNTs and was the largest among the tensile strengths of all the N-MWCNT-based actuators tested. It also had the highest thermal stability among all the actuators, both with and without MWCNT addition. In addition, the blocking force and displacement of the N-MWCNT 0.2 wt%-based actuator were 0.086 mN and 0.0845 mm, which are 1.6 and 2.34 times higher, respectively, than those of the actuator without MWCNTs. As a result, the addition of MWCNTs afforded the actuator with good mechanical and chemical properties, including high strength and thermal stability of the Nafion membrane. However, if the MWCNT weight ratio exceeds the suitable amount, sufficient dispersion is not achieved, which can decrease the membrane mechanical and chemical properties. Finally, the developed HPTS method can effectively be used in research on polymer materials that requires controlled thicknesses, and the N-MWCNT 0.2 wt%-based actuator can be applied to flexible devices, artificial robots, and flexible actuator technologies.
